# The transtheoretical model (TTM) questionnaire for smoking cessation: psychometric properties of the Iranian version

**DOI:** 10.1186/1471-2458-13-1186

**Published:** 2013-12-17

**Authors:** Fatemeh Sarbandi, Shamsaddin Niknami, Alireza Hidarnia, Ebrahim Hajizadeh, Ali Montazeri

**Affiliations:** 1 Department of Health Education, Faculty of Medical Sciences, Tarbiat Modares University, Jalal Ale Ahmad Highway, P.O. Box: 14115-331, Tehran, Iran; 2 Department of Biostatistics, Faculty of Medical Sciences, Tarbiat Modares University, Jalal Ale Ahmad Highway, P.O. Box: 14115-331, Tehran, Iran; 3 Mental Health Research Group, Health Metrics Research Center, Iranian Institute for Health Sciences Research, ACECR, P.O. Box 13185-1488, Tehran, Iran

## Abstract

**Background:**

The transtheoretical model (TTM) is a common framework for studies of smoking cessation. Using the TTM, several instruments were developed to measure to what extent interventions could make changes in people’s behavior. The current study aimed to test the validity and reliability of the Persian version of a TTM based questionnaire for smoking cessation in Iran.

**Methods:**

This was a cross-sectional validation study among adult male smokers using the TTM Questionnaire. Backward-forward procedure was applied to translate the questionnaire from English into Persian (the Iranian language). The confirmatory factor analyses were performed to test validity. The internal consistency and stability of the questionnaire was examined using Cronbach’s alpha coefficient and Intraclass Correlation Coefficient (ICC).

**Results:**

In all 150 male smokers were entered into the study. The mean age of participants was 36.51 ± 7.94 years. The results obtained from confirmatory factor analysis revealed that the data was fit to the model: the goodness of fit index (GFI) = 0.92; the comparative fit index (CFI) = 0.91; the root mean square error of approximation (RMSEA) = 0.065 (95% CI = 0.063-0.067), and the relative chi-square (x^2^/df) = 1.87, p < 0.001. The Cronbach’s alpha ranged from 0.60 to 0.84 indicating an acceptable result. Also Intraclass Correlation of Coefficient (ICC) ranged from 0.61 to 0.83 corresponding to a satisfactory finding.

**Conclusion:**

The current study provided psychometric evidence for an appropriate, reliable, and valid tool to determine smoking behaviors among Iranian adult smokers. Indeed the findings from this study could be applied in designing smoking cessation interventions in Iran.

## Background

**Figure 1:**
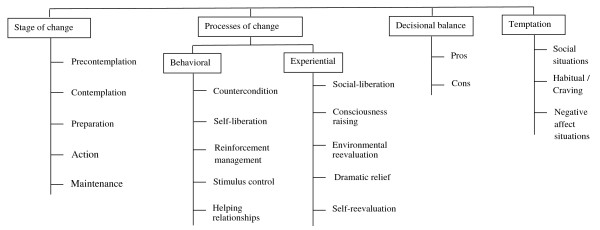
Schematic view of the transtheoretical model (TTM) constructs.

Successful strategies implementing smoking cessation are account for cardinal investments in tobacco control programs [1]. Such strategies are theory driven and usually are based on various models of behavioral change. One of the models that have been utilized in smoking cessation is the transtheoreticlal model (TTM). The TTM emerged from more than 300 theories of psychotherapy and behavior change that has been validated and popularized by Prochaska and colleagues since over last 20 years [2]. The TTM consists of four constructs namely: stage of change, processes of change, decisional balance, and temptation [2-4]. In fact this model offers a framework that assumes health behavior change involves progress through five stages of change, ten processes of change, two decisional balance and three temptation domains as summarized in Figure 1.

Using the TTM several instruments were developed to measure to what extent interventions could make changes in people’s behavior. For instance there are instruments that measure life style changes such as physical activity, nutrition, weight loss, and smoking [4-9]. In western countries the English version of the TTM Questionnaire(s) have been validated in many investigations [9-12], but unfortunately in developing countries the literature on the topic is limited. An instrument that describes health behavior or health behavior changes can be used in different cultures and ethnic backgrounds if ensuring that essential linguistic adaptation was applied. In fact culturally and linguistically competent questionnaires take into account cultural values, beliefs and practices that might differ among different populations. Therefore, it is necessary to re-examine the validity and reliability of such measures in any given culture. Hence, the aim of this research was to test the validity and reliability of the Persian version of TTM Questionnaire for smoking cessation in Iran in order to provide a theory-based instrument for measuring behavior changes following public health interventions.

## Methods

### Participants and procedures

This was a cross-sectional validation study conducted between November and December 2012. Participants were recruited from 6 factories in Tehran, Iran. Inclusions criteria were; being male, current smoker with history of smoking at least 100 cigarettes, not involved in any quit attempt, planning on quitting smoking in the next 30 days, and being able to read and write in Persian. The main reason for including only one group (people in the preparation stage) was due to the fact that we were planning to implement an intervention for these people. Since according to the TTM, every stage of change needs its own strategy for any possible interventions [2]; we thought the most convenient sample for the study would be a sample of individuals who are at preparation stage; otherwise we should have been implemented a number of different interventions for people in different stages of change and this was impossible for several reasons including time constrains and scarce resources. However, the study questionnaire was distributed to the participants at worksite settings over a period of two weeks.

### The TTM questionnaire

The TTM Questionnaire for smoking cessation was developed by Prochaska and colleagues [12,13] and it is available in two versions: the original questionnaire containing 83 items and the short form containing 38 items. In the current study we used the short form. Both versions include 4 constructs:

a. The stage of change: that assesses the current conditions of individuals’ smoking habits and whether they wish to quit smoking or not. In theory there are five stages and an individual could indicate that he or she is at one of these stages that are: precontemplation, contemplation, preparation, action and maintenance. For instance first it distinguishes current smoker, ex-smoker and nonsmoker. Then current smokers answer two questions: history of quit smoking and cessation stage of smoking. In this study if a participant was planning to quit smoking within the next 30 days and had made a quit attempt in the past year he was identified as being in preparation stage. The stage of change is a dependent variable and does not incorporate into the model [9-11,14].

b. The processes of change: it measures ten processes of change under two main categories: experiential and behavioral activities to change their smoking behavior. It includes 10 statements about experiential and 10 statements about behavioral processes. Examples of items for measuring each ‘processes of change’ are displayed in Table 1. In the version used here, participants responded to each item on a five-point Likert scale (1 = never; 5 = repeatedly).

c. Temptation: it is a tool for assessing situational temptation. Three sub-factors were used in this study. This construct contains three items on social situations, negative affect situations, and habitual/craving situations. The response categories range from 1 (not at all tempted) to 5 (extremely tempted).

d. Decisional balance: it measures smoker’s opinion on quitting. This construct contains six items including items on pros and cons. The items employed a five-point Likert-type scale ranging from 1, (not important) to 5 (extremely important).

**Table 1:** Processes of change used in the study

**Sub-factors**	**Behavioral/experiential process**	**Item example**
Counterconditioning	Behavioral	When I am tempted to smoke I think about something else
Self-liberation	Behavioral	I tell myself I can quit if I want to
Social-liberation	Experiential	I notice that nonsmokers are asserting their rights.
Consciousness raising	Experiential	I recall information people have given me on the benefits of quitting smoking
Reinforcement management	Behavioral	I am rewarded by others if I don’t smoke.
Environmental reevaluation	Experiential	I consider the view that smoking can be harmful to my environment
Dramatic relief	Experiential	Warnings about the health hazards of smoking move me emotionally
Self-reevaluation	Experiential	I get upset when I think about my smoking.
Stimulus control	Behavioral	I remove things from my home or place of work that remind me of smoking
Helping relationships	Behavioral	I have someone who listens when I need to talk about my smoking

### Translation

The TTM Questionnaire was translated from English into Persian by two translators and back translated by two independent translators. The translators were fluent in both English and Persian. They were all experienced health care professionals who have been working for many years. Then, the research team and translators examined the questionnaire for accuracy [15]. To assess the content validity a panel of experts including 10 health professionals (seven specialists in health education and three experts in tobacco control) evaluated the questionnaire for technical issues and wordings. Item allocation and scaling also was checked. Accordingly a few minor changes were made. The face validity of the instrument was assessed by 20 male smokers to insure that they understood questions and had no difficulties in responding to questions [16]. This sample was not included in the main study.

### Statistical analysis

Descriptive statistics were computed for describing the characteristics of the sample. In order to examine the psychometric properties of the questionnaire several statistical tests were applied:

Validity: The confirmatory factor analysis (CFA) was performed in order to test the assumed theoretical framework behind the instrument. Various fit indices were used to asses fit of the model to the data: the goodness of fit index (GFI), the comparative fit index (CFI), the root mean square error of approximation (RMSEA), and the relative chi-square statistic (x^2^/df). Value of the GFI and the CFI around 0.90 were considered acceptable [17,18]. For the RMSEA and the relative chi-square values of ≤ 0.08 and ≤ 3 reflected acceptable fit of the model, respectively [18-21].

Reliability: to determine the reliability of the instrument, the internal consistency was tested using the Chronbach’s alpha coefficient. We also estimated intraclass correlation coefficient (ICC) in order to assess the stability. Forty smokers from the same sample were randomly selected and responded to the questionnaire twice with a 2-weeks interval. The statistical program SPSS for Windows version 16.0 and LISREL 8.80 was used to carry out the analyses. The P value was set at 0.05.

### Ethics

The ethics committee of Trabiat Modares University approved the study. Informed consent was obtained from all participants.

## Results

In all 150 smokers were entered into the study. The mean age of participants was 36.51 ± 7.94 years ranging from 22 to 58. The mean age of initiation of smoking was 19.29 ± 5.27 year. The characteristics of the study sample are shown in Table 2.

**Table 2:** Demographic characteristics of the study sample (n = 150)

	**Number**	**%**
**Age**		
22-30	38	25.3
31-38	58	38.7
39-46	32	21.3
47-58	22	14.7
Mean (SD)	36.51 ± 7.94	-
**Marital status**		
Single	23	15.3
Married	127	84.7
**Education**		
< 12 years	52	34.7
12 years	66	44/0
> 12 years	32	21.3
**Fagerstrom test for nicotine dependence**		
≤ 5	126	84.0
> 5	24	16.0

### Validity

**Figure 2:**
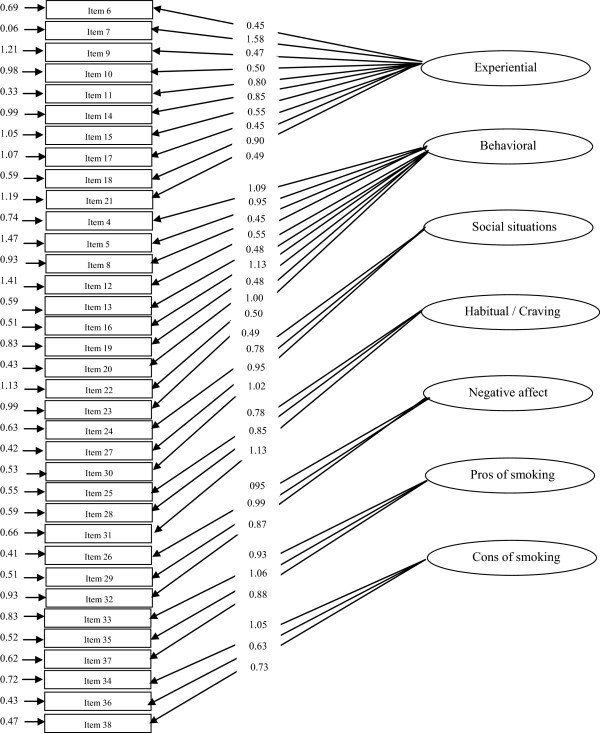
**The results obtained from the confirmatory factor analysis (items are observed variables and subscales are latent variables.** (The arrows connect subscales and its items with their correlation).

The confirmatory factor analysis was performed and the following fit indices were found: the goodness of fit index (GFI) = 0.92; the comparative fit index (CFI) = 0.91; the root mean square error of approximation (RMSEA) = 0.065 (95% CI = 0.063-0.067), and the relative chi-square (x^2^/df) = 1.87, p < 0.001. The model fitted exactly for the study population. However to improve the model fit, modification indices for the regression weights were examined in order to specify covariances among the indicators or factors. Since a remarkable improvement on fit indexes was not observed, no modifications were made. And thus the model was accepted in its current form. The results are presented in Figure 2.

### Reliability

After the confirmatory factor analysis, the internal consistency of the questionnaire as measured by the Cronbach’s alpha was evaluated. Alpha coefficient applied separately for all seven factor ranged between 0.60 and 0.84, which are accepted value. In addition the stability of the instrument as measured by intraclass correlation of coefficient (ICC) was satisfactory. The results are given in Table 3.

**Table 3:** The internal consistency and stability of the questionnaire as measured by Cronbach’s alphas and intraclass correlation coefficient (ICC)

	**Number of items**	**Cronbach’s alphas**	**ICC**
**Processes of change**	**20**	**0.79**	**0.70**
Behavioral	10	0.72	0.77
Experiential	10	0.69	0.71
**Temptation**	**9**	**0.82**	**0.90**
Social situations	3	0.75	0.78
Habitual/craving	3	0.80	0.83
Negative affect situations	3	0.84	0.79
**Decisional balance**	**6**	**0.60**	**0.71**
Pros of smoking	3	0.79	0.82
Cons of smoking	3	0.60	0.61
**Total**	**35**	**0.80**	**0.65**

Figures in bold are calculated for Process of change, Temptation, Decisional Balance, and for the all items.

## Discussion

The present study performed a rigorous psychometric evaluation for the translated Iranian version of the short form TTM Questionnaire for smoking cessation. In general, the result showed that it was a valid instrument for measuring smoking behavior change among Iranians. Developing theory-based instruments are considered an important prerequisite for any attempt to implement and evaluate health education/promotion interventions. Therefore in this context we believe the findings from the current study could be helpful for those who are involved in tobacco control programs both at action level and at research settings.

We performed the confirmatory factory analysis in order to ascertain if the coherence between the data and the theoretical structure exist. The results indicated that the instrument included seven subscales. Of these, two subscales meant to refer to the processes of change [22]. The process of change is a key construct of the TTM and thus merits full consideration when developing instruments for assessing behavior changes. The contribution of such subscales to the process of change implies when developing targeted interventions one should be careful to provide conditions to move target audience from preparation to action stage.

Temptation reflects a psychological state that might encourage an individual to engage in smoking when confronted with a difficult situation. As expected, the nine items of the temptation construct were organized three subscales: ‘social situation’, ‘habitual/craving’ and ‘negative affect’. This result replicates the structure found in other studies [23-26]. In fact, this model suggests that the three subscales should be considered when designing a tailored intervention for smoking cessation.

As expected, decisional balance revealed two subscales: pros and cons [23,25,27]. This result indicates that the instrument was well discriminated benefits and barriers involved in making the decision to quit smoking. Decisional balance of the TTM implies that pros and cons are relatively important part of the model. Perhaps for changing risk behaviors among target populations it is essential to focus on pros and cons for that specific behavior.

The findings indicated that the model from the original inventory produced an acceptable fit to our data [28]. In addition we performed two statistical tests to assure that the instrument was reliable. The Cronbach’s alphas coefficient for the instrument indicated that all measures had good internal consistency [29]. Also test-retest reliability was carried out as a measure of instrument’s stability [30]. The findings were satisfactory lending support for its further reliability.

### Limitations

This study had some limitations. The study participants were male and it is not clear if we included females in the study we would obtain the same results. Furthermore, we recruited smokers who were in the preparation stage and thus the reliability and validity of the measure may look different among smokers in other stages. Nevertheless, the findings from this study showed that this instrument could be used for measuring behavior changes in interventions designed for quit smoking among adult male who wish to change his behavior in the next 30 days.

## Conclusion

The current study provided psychometric evidence for an appropriate, reliable, and valid tool to determine smoking behaviors among Iranian adult smokers. This instrument can be utilized for behavior change studies. Indeed, the findings from this study could be applied in designing smoking cessation interventions.

## Competing interests

The authors declare that they have no competing interests.

## Authors’ contributions

FS was the main investigator, designed the study, collected the data and wrote the first draft. SN supervised the study. AH and EH were the study consultants. AM critically reviewed the paper and provided the final manuscript. All authors read and proved the final draft.

## Pre-publication history

The pre-publication history for this paper can be accessed here:

http://www.biomedcentral.com/1471-2458/13/1186/prepub
